# Immune dysregulation in prolonged Long-COVID: lymphocytes emerge as key mediators of persistent inflammation, exhaustion and cytotoxicity

**DOI:** 10.1186/s12967-026-08081-6

**Published:** 2026-04-11

**Authors:** Marta Liva Springe, Kristīne Vaivode, Rihards Saksis, Nineļa Miriama Vainšeļbauma, Laura Ansone, Monta Brīvība, Helvijs Niedra, Vita Rovite

**Affiliations:** Latvian Biomedical Research and Study Centre, Riga, LV-1067 Latvia

**Keywords:** COVID-19, Long-COVID, Single-cell RNA sequencing, Immune landscape, Lymphocyte, Exhaustion, Cytotoxicity, Interferon, Intercellular communication

## Abstract

**Background:**

Long-COVID affects at least 10% of COVID-19 survivors, displaying debilitating symptoms across multiple organ systems. Despite the widespread prevalence, Long-COVID aetiology remains poorly understood, but emerging evidence points to immune dysregulation as a potential mechanism involved in its development or persistence.

**Methods:**

This study presents a unique analysis of the peripheral blood mononuclear cell transcriptomic profile of COVID-19 and Long-COVID patients at single-cell resolution. We reconstructed the cell state and intercellular communication using differentially expressed gene profiling and ligand–receptor interaction analyses.

**Results:**

Our results reveal altered T and natural killer cell subset proportions, diminished proliferating lymphocyte and B cell signalling capacity, and the expression of exhaustion and cytotoxicity associated genes 1.5–2 years post-infection, suggesting incomplete immune recovery. Distinct interferon responses in these cell populations at the acute phase for patients who go on to develop Long-COVID indicate early disease mediator potential.

**Conclusions:**

Collectively, these findings provide insight into the immune processes underlying the progression of COVID-19 into a chronic Long-COVID state. The observed changes in immune cell subsets at the acute phase of the infection may be predictive of Long-COVID progression and could be useful in understanding disease aetiology while the observed long-term effects are crucial to developing therapeutic and diagnostic tools.

**Supplementary Information:**

The online version contains supplementary material available at 10.1186/s12967-026-08081-6.

## Background

Severe acute respiratory syndrome coronavirus 2 (SARS-CoV-2) emerged in late 2019 and by March 11, 2020 was declared the causative agent of a global pandemic of acute respiratory disease designated coronavirus disease 2019 (COVID-19) [1]. This pandemic tragically resulted in over 7 million deaths and affected the long-term health of millions of survivors [2]. The virus is responsible for a plethora of symptoms, including chest pain, shortness of breath, and cognitive dysfunction, that may persist or even develop after the initial COVID-19 infection has been resolved [3, 4]. Commonly referred to as ‘Long-COVID’, other umbrella terms such as ‘Post-COVID-19 condition’, ‘Post-acute COVID syndrome’, and ‘Post-acute sequelae of SARS-CoV-2’ are often used interchangeably to describe the various symptoms persisting for at least 3 months after acute COVID-19 infection, with no other underlying cause [5].

It is generally accepted that Long-COVID develops in 10–20% of COVID-19 survivors [5]. However, recent studies have shown that the general incidence of Long-COVID may have been underestimated and that the condition disproportionately affects different groups of the population [6–11]. Several studies have shown that women are at higher risk of developing Long-COVID [6, 11]. Severe acute COVID-19 also considerably increases the likelihood of developing Long-COVID [11]. Furthermore, smoking, older age, and diabetes mellitus have been cited as risk factors [12]. Conflicting evidence has emerged regarding the potential associations between Long-COVID and vaccination history [9, 13, 14] or the virus variant [4, 12], which require further study.

The symptoms associated with Long-COVID range from mild to severe, with severe symptoms being profoundly debilitating for many patients [15]. Both acute COVID-19 and Long-COVID symptoms manifest across multiple organ systems; notably, pulmonary and cardiovascular complications pose a serious threat to the life and health span of patients [3, 4, 16]. Beyond the known incidence of patients presenting respiratory distress and cardiovascular events during the acute phase of COVID-19, an increase in a range of associated complications in the cases that develop Long-COVID, including myocarditis, dyspnoea, and the requirement for oxygen support, has also been observed [3, 4, 16]. Research indicates the involvement of immune system dysfunction during COVID-19, potentially leading to prolonged dysregulation and contributing to lasting complications in Long-COVID cases [17].

Characterizing the dynamic immune response and functional alterations of T cells in COVID-19 has been essential to understanding the virus–host immune system interactions [18, 19]. Recently, specific subsets of CD8^+^ T cells expressing PD1 and CTLA4 were detected in Long-COVID cases 8 months post-acute infection [17]. These markers are associated with T cell exhaustion, which is driven by chronic antigen stimulation and is known to play a role in persistent viral infections [18, 19]. Furthermore, severe COVID-19 has been shown to induce lasting effects on the function of the innate immune system. Monocyte populations in Long-COVID peripheral blood mononuclear cells (PBMCs) have been described as dysfunctional [20], hyper-responsive [21], or otherwise altered [22]. However, their precise role in driving Long-COVID pathogenesis and severe COVID-19 states remains unclear. Several different immune mechanisms underlying lasting COVID-19 complications have been studied, yet whether these are involved in promoting, sustaining, or aggravating Long-COVID remains to be elucidated. Building on previous findings, including our prior study [23] in which we observed increased numbers of immature neutrophils in the PBMCs of a Long-COVID patient 3 months post-infection and a large population of mature neutrophils at 2 years post-infection, we aim to investigate these immune alterations temporally in a larger patient cohort in the current study.

Given the sudden onset and rapid spread of SARS-CoV-2, it is crucial to study the long-term effects and implications of this virus infection of the life and health span of patients. The number of Long-COVID cases continues to rise, and the number of patients requiring long-term post-COVID-19 care is also increasing [4]. It is therefore crucial to conduct in-depth follow-up with patients, study the symptoms presenting after acute infection resolution, and investigate the mechanisms driving systemic Long-COVID conditions.

To further investigate the involvement of immune cells in acute and Long-COVID-19, we conducted single-cell RNA sequencing (scRNA-seq) for the characterization of PBMCs from a select cohort of patients during acute COVID-19 infection, and at 3 months and 1.5–2 years post-acute infection. We obtained blood samples from female patients who had been hospitalized during acute infection and established three study groups: patients who later developed Long-COVID with lasting cardiovascular complications (hereafter referred to as LC-CV) or lasting pulmonary complications (LC-Pulm), and patients who did not develop Long-COVID (Non-LC). Specifically, we focused on female patients with severe initial infection, as sex and disease severity have been recognized as risk factors for developing Long-COVID [6, 11]. ScRNA-seq allows for the identification of individual cells, the classification of cell subsets, and analysis of the cellular interactions involved, which may provide insights into the pathophysiological principles of Long-COVID. Our cohort is distinguished by deep clinical phenotyping and long-term follow-up, which together provide substantial interpretative value despite the modest sample size. The assembly of comparable cohorts with similar longitudinal depth and high-resolution molecular profiling is logistically demanding and resource-intensive, and such datasets remain rare in the field. Only a limited number of studies have attempted comprehensive characterization of the broad spectrum of post-COVID sequelae within the months after infection [24], and detailed immune profiling at extended follow-up time points is even less common. The aim of our study was to unravel the intricate details of immune cell transcriptomic alterations and the immune system perturbations involved in acute and chronic COVID-19 conditions in a longitudinal manner.

## Materials and methods

### Overview of the patient cohort

This prospective study involved female patients who had been hospitalized with acute COVID-19 (average age: 55, median: 52; average BMI: 28.22, median: 27.72) at Riga East University Hospital, Latvia between October 2020 and January 2021. Written informed consent was obtained from every participant before their inclusion in the study. The study was approved by the Central Medical Ethics Committee of Latvia (No. 01–29.1.2/928) and was carried out according to the Declaration of Helsinki and the Department of Health and Human Services Belmont Report. COVID-19 infection was confirmed by an RT-PCR test, and blood sampling was arranged at the acute infection phase, at 3 months, and between 18 and 24 months post-acute infection. Peripheral blood samples were obtained using the BD Vacutainer® CPT™ (USA). At 18–24 months post-acute COVID-19, patients who displayed Long-COVID with cardiovascular (LC-CV, *n* = 3, average age: 54, median: 52; average BMI: 34.56, median: 33.09) and pulmonary complications (LC-Pulm, *n* = 3, average age: 65, median: 68; average BMI: 24.56, median: 23.88) along with patients who did not develop Long-COVID (Non-LC, *n* = 3; average age: 47, median: 47; average BMI: 25.57, median: 27.72), were selected for this study. All patients were non-smokers, and all except one patient in each complication group (LC-CV and LC-Pulm) had been vaccinated with the Pfizer-BioNTech, Moderna, or Jannsen vaccine after resolution of the initial infection (Table S1).

### Sample preparation and storage

Peripheral blood samples were collected using BD Vacutainer® CPT™ collection tubes according to the manufacturer’s instructions and processed less than 2 hours after sample collection to preserve cell quality. All safety measures when working with human samples and pathogens were taken into consideration throughout the study. Mononuclear cells were then isolated using the density gradient centrifugation method, counted using Trypan blue (Thermo Fisher Scientific), and stored in foetal bovine serum (Thermo Fisher Scientific) with 10% dimethyl sulfoxide (Sigma-Aldrich) in liquid nitrogen.

### Single-cell RNA library preparation

Single-cell suspensions were washed twice with phosphate-buffered saline containing 0.04% bovine serum albumin (Sigma-Aldrich), cells were counted, and 30 000 cells were used for further analysis with the DNBelab C Series High-throughput Single-cell RNA Library Preparation Set V2.0 (MGI, Shenzhen, China). In short, single cells were combined with magnetic beads and encapsulated in oil droplets using the DNBelab C4 microfluidic device for mRNA hybridization. Magnetic beads were collected, mRNA from single cells was reverse transcribed, and libraries were prepared according to the manufacturer’s protocol.

The resulting cDNA product fragment size was between 1149 bp to 1492 bp. cDNA and oligo libraries had fragment sizes between 428 bp to 515 bp and 181 bp to 190 bp, respectively. Quality control of fragment size and concentration was performed using the Agilent 2100 Bioanalyzer System and the Qubit dsDNA HS Assay kit for the Qubit® 2.0 Fluorometer (Thermo Fisher Scientific).

### Sequencing

Sequencing was carried out using the DNBSEQ-G400RS platform (MGI). Prepared libraries were circularized and DNA NanoBalls (DNBs) were generated according to the manufacturer’s protocol. The resulting DNBs were loaded onto the sequencing flow cell and sequenced with the DNBSEQ-G400RS High-throughput Sequencing kit FCL PE100 (MGI) according to the manufacturer’s instructions.

### Data analysis

Raw sequence data processing, including primary quality control, alignment, annotation, and quantification, was performed using DNBelab C Series HT scRNA analysis software (*v2.1.1*) [25]. Within this pipeline, the sequence reads were aligned to the GENCODE v44 [26] *Homo sapiens* reference genome. The filtered expression matrices and cell barcodes were then used for downstream analysis in the R [27]/RStudio (*v4.4.2*/*v2023.12.1*) [28] environment using the Seurat (*v5.1.0*) [29] workflow.

The downstream analysis consisted of two parts. After importing the data into the Seurat ecosystem, a dataset of 39 349 genes across 223 087 cells was generated. Following the analysis described below, 44 369 cells in three clusters were identified, for which specific cell populations could not be detected due to their low overall expression levels and the lack of specific marker signatures for the known immune cell types. These cells were removed from the data set, and all the initial data analysis steps were repeated. The remaining 178 719 cells were analysed further.

We initially examined the distribution of multiple cell and gene quality indicators, excluding cells based on the following criteria: <500 genes, a mitochondrial gene ratio > 20%, a novelty score (log_10_ genes per unique molecular identifier) <85%, and <500 or > 5 000 unique molecular identifiers. Subsequently, genes that were expressed in < 10 cells were also excluded. This additional quality filtering resulted in 38 280 genes across 174 336 cells.

Next, we scaled the data and selected the 3 000 most variable features using the ‘vst’ selection method. Using the principal component analysis (PCA) method, we calculated the cell cycle scores and evaluated the cell grouping across multiple variables to select the regression variables for normalization. SCTransform was used for sample level normalization, with the mitochondrial ratio, cell cycle S, and G2M scores used as the regression variables.

Next, we selected 3 000 integration features for PCA ordination, then integrated the SCT data using Harmony (*v1.2.0*) [30] and PCA as the reduction method, with the Long-COVID complication groups and time points as covariates. Based on the elbow plot generated using the cumulative variation in the principal components, we selected the first 30 principal components for uniform manifold approximation and projection (UMAP) dimension reduction. We also determined the clusters at different clustering resolutions (0.2–2.0) using the shared nearest neighbour method, from which a resolution of 0.8 was selected, as it provided a reasonable number of clusters together with good visual separation when evaluating the UMAP plot. The resulting cluster resolution yielded 22 cell clusters.

### Cell type annotation

To determine the cell types in our dataset, the ScType (*v1.0*) [31] approach was employed that uses a precompiled list of cell type markers for multiple cell types. Using this approach, we obtained a preliminary list of cell populations present in our dataset. Next, we evaluated the ScType results and used the Seurat’s FindAllMarkers method with the Wilcoxon test to obtain a list of differentially expressed markers (min. pct = 0.25, log_2_ fold change > 0.25) between the determined cell clusters along with our own list of cell markers. The differentially expressed markers were examined using a DotPlot graph for their prevalence among the cells in each cluster and their expression levels. Finally, we obtained a list of 19 unique cell populations for the 22 clusters detected. To determine the differences in cell counts between different time points and complication groups for the detected cell types, we used a cell proportion test from the scProportionTest (*v0.0.0.9000*) [32] package and proportionally transformed cell count bar plot graphs for each cell type.

Cell exhaustion and cytotoxicity states were defined for proliferating lymphocyte, T cell, and natural killer (NK) cell subpopulations using well-established marker genes associated with either cell exhaustion (*TIGIT*, *PDCD1*, *HAVCR2*, *TOX*, *IRF4*, *LAG3*, *BTLA*, *VSIR*, *CD96*, *CD28*, *CD226*) or cytotoxicity (*IFNG*, *GNLY*, *GZMA*, *GZMH*, *KLRK1*, *KLRB1*, *CTSW*, *CST7*). The AddModuleScore function was used in Seurat to establish a score for each T, NK, B, and monocyte cell subset indicating the interferon (IFN)-α/β/γ response, inflammatory response, apoptosis, and migration scores, using the RESPONSE TO INTERFERON ALPHA (GO:0035455), RESPONSE TO INTERFERON BETA (GO:0035456), RESPONSE TO INTERFERON GAMMA (GO:0034341), ACUTE INFLAMMATORY RESPONSE (GO:0002526), APOPTOTIC SIGNALING PATHWAY (GO:0097190), and LEUKOCYTE MIGRATION (GO:0050900) gene sets, respectively. The exhaustion scores were compared using the Kruskal–Wallis omnibus test to evaluate the presence of overall differences and the pairwise Dunn’s test with Holm–Bonferroni *p*-value adjustment from the rstatix (*v0.7.2*) [33] package to obtain results for individual pairs of comparisons. The results were visualized in the form of box plots generated using the ggplot2 (*v3.5.1*) [34] and ggpubr (*v0.6.0*) [35] packages.

### Differential gene expression

Differential expression analysis between cell types of interest in the Non-LC and Long-COVID cohorts, as well as in the Non-LC versus the pulmonary (LC-Pulm) and cardiovascular (LC-CV) complication groups separately, was performed using Seurat’s FindAllMarkers function with the Wilcoxon test option and the cut-off values of p-adj < 0.05 and log_2_ fold change ≥ 0.25. Only genes expressed in at least 10% of cells in the cell group were considered. Upon completion of differential expression analysis, the results were visualized in volcano plots using the EnhancedVolcano (*v1.24.0*) package [36].

### Differential intercellular communication inference with CellChat

To provide a more systems-level approach to studying the cellular dysregulation associated with Long-COVID, the CellChat (*v2.1.1*) [37] program was used to infer cell-to-cell communication patterns and the changes in these patterns between the complication and control groups from scRNA-seq gene expression data. CellChat employs an extensive database of ligand–receptor interactions (CellChatDB) combined with mass-action-based models for statistical inference of communication probabilities between cell groups that adjusts for cell population size. With this communication probability inference, CellChat represents the scRNA-seq dataset as a network of interacting cell groups, which can then be projected onto the human protein-to-protein interaction network for more accurate results.

In this study, the Secreted Signalling database from CellChatDB, which focuses on cell-to-cell communication through cytokine and chemokine signalling, was used. The integrated Seurat object of Long-COVID and Non-LC patient cells was converted into separate CellChat objects for each time point and condition. In accordance with the CellChat guidelines, overexpressed genes were identified at the threshold of *p* < 0.05, and expression data were projected onto the human protein-to-protein interaction network. Cell communication probabilities were calculated with the triMean type of average per-cell group expression estimation and filtered at the threshold of at least 10 cells in each communicating group. Based on the calculated probabilities, cell-to-cell communication networks were then constructed and aggregated. Long-COVID and Non-LC CellChat objects were merged for each visit and cellular communication analyses were performed following CellChat guidelines. Differential interaction number and strength per pair of cell types between the patient groups were visualized as a heatmap (Fig. S2, Fig. 3C) using the netVisual_heatmap function. The changes in total mean interaction number and interaction strength were also compared between the Long-COVID and Non-LC patient groups at the acute phase and 1.5–2 years post-infection using a two-tailed Wilcoxon rank-sum test (*p* < 0.05 significance level).

## Results

### Single-cell RNA sequencing uncovers unique cell types in COVID-19 and prolonged Long-COVID patient PBMCs

**Fig. 1:**
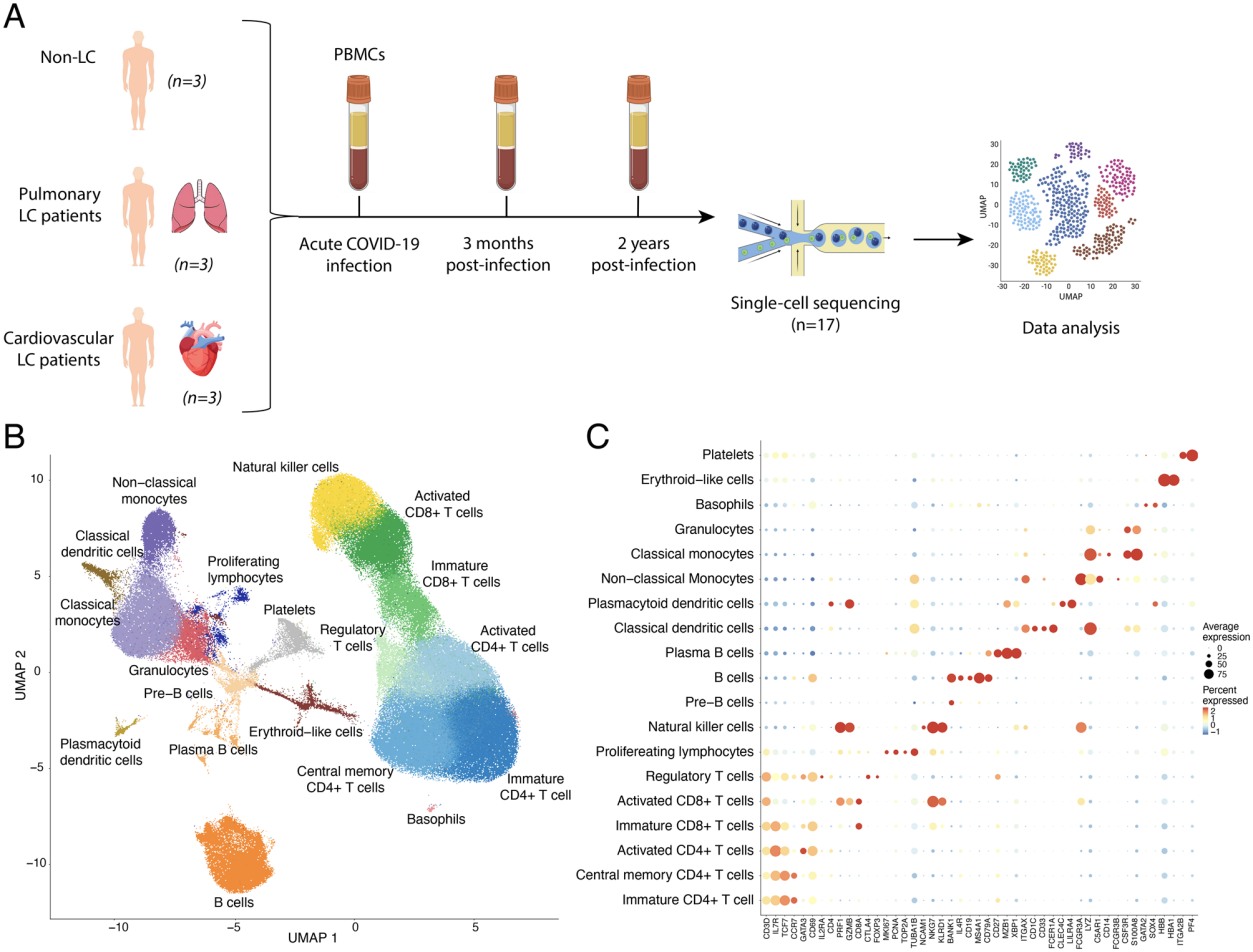
Study design and cell population identification. (**A**) Overview of the study design. The workflow consists of PBMC isolation using blood samples from 9 patients, single-cell RNA sequencing, and data analysis. Samples were obtained from patients during the acute phase of infection, after 3 months, and after 1.5–2 years. Patients who developed cardiovascular (LC-CV, *n* = 3) and pulmonary (LC-Pulm, *n* = 3) Long-COVID complications were selected for the study. Patients who did not develop Long-COVID (Non-LC, *n* = 3) were used as controls. (**B**) Integrated single-cell population landscape across all conditions and samples. The UMAP projection shows a total of 174 336 cells classified into 22 clusters and 19 cell populations. Each point represents a single cell coloured according to the inferred cell type. Cell type identification based on ScType [31] and gene marker average expression and prevalence, as shown in panel C. (**C**) Gene expression markers used to differentiate cell populations and subpopulations. The dot plot shows the identified cell populations along the y-axis and marker genes along the x-axis. Dot size indicates the average expression of the marker gene in each population. Dot colour indicates the percentage of cells in each population in which the marker gene was expressed

To investigate the immune cell landscape of patients with acute COVID-19 infection, those who had fully recovered, and those who had developed Long-COVID (LC), we implemented scRNA-seq on PBMCs. We obtained RNA transcript profiles at single-cell resolution and filtered for high-quality cells, resulting in a total of 174,336 cells from 9 patients. Each patient was classified as recovered (Non-LC, *n* = 3), cardiovascular Long-COVID (LC-CV, *n* = 3), or pulmonary Long-COVID (LC-Pulm, *n* = 3). The study design is depicted in Fig. 1A. All patients were hospitalized during the acute phase of COVID-19 infection. Blood samples were obtained from these patients during the acute phase, after 3 months, and 1.5–2 years post-infection (Fig. 1A).

Results of the cell analysis were presented in a UMAP graph showing 22 cell clusters, representing proximity in gene expression across individual cells (Fig. 1B). Clusters were identified as cell types according to ScType [31] and well-established marker genes (Table 1), as shown in Fig. 1C. A total of 19 cell-type clusters were identified, representing blood immune cells (Table 1, Fig. 1C).

**Table 1:** Marker genes used for cell type identification

Cell type	Marker gene
Immature CD4^+^ T cell	*CD3D, IL7R, TCF7*
Central memory CD4^+^ T cells	*CCR7*
Activated CD4^+^ T cells	*GATA3, CD69, IL2RA*
Immature CD8^+^ T cells	*CD4, PRF1*
Activated CD8^+^ T cells	*GZMB, CD8A*
Regulatory T cells	*CTLA4, FOXP3*
Proliferating lymphocytes	*MKI67, PCNA, TOP2A, TUBA1B*
Natural Killer cells	*NCAM1, NKG7, KLRD1*
Pre-B cells	*IL4R*
B cells	*CD19, MS4A1, CD79A*
Plasma B cells	*CD27, MZB1, XBP1*
Classical dendritic cells	*ITGAX, CD1C, CD33, FCER1A*
Plasmacytoid DCs	*IL3RA, CLEC4C, LILRA4*
Non-classical Monocytes	*FCGR3A*
Classical Monocytes	*LYZ, C5AR1, CD14*
Granulocytes	*CSF3R, FCGR3B, S100A8*
Basophils	*IL3RA*
Erythroid-like cells	*HBB, HBA1*
Platelets	*ITGA2B, PF4*

### Distinct immune cell population distribution among patients with Long-COVID

**Fig. 2:**
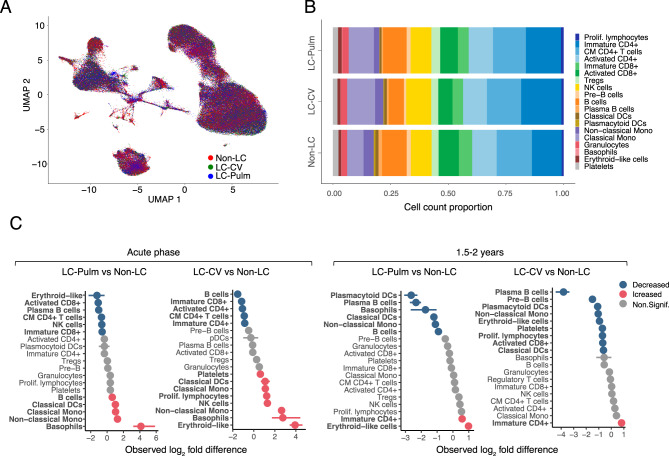
Proportional differences in identified cell populations across conditions and time points. (**A**) Overlay of the single cell landscape across three conditions: patients who did not develop Long-COVID (Non-LC, *n* = 3), patients who developed Long-COVID with cardiovascular (LC-CV, *n* = 3) or pulmonary (LC-Pulm, *n* = 3) complications, shown as a UMAP projection. Each point represents a cell, coloured according to its respective sample group. (**B**) Distribution of cell population proportions in each of the three conditions. Sample complication groups are displayed along the y-axis, and cell count proportions are displayed along the x-axis. (**C**) Relative cell numbers in each cell population, compared between the LC-Pulm and Non-LC groups, and between the LC-CV and Non-LC patient groups at two time points: the acute phase of COVID-19 infection and 1.5–2 years post-infection, expressed as a log_2_ difference

We further examined the differences in immune cell populations between patient groups based on the identified clusters. The UMAP overlay of the immune cell landscapes of the Non-LC, and LC-Pulm and LC-CV complication groups showed the differential cell clustering between disease conditions (Fig. 2A). Of note, proliferative lymphocytes displayed unique clustering patterns between the three groups (Fig. S1). The patient cell landscape between the three timepoints (acute phase, 3 months, and 1.5–2 years post-infection) is depicted in Fig. S2. Figure S3 represents each of the patient groups individually at acute COVID-19 infection, 3 months and 1.5–2 years post-infection.

Although UMAP visualization showed substantial overlap across conditions (Fig. 2A), clustering patterns can be further emphasized by observing the relative abundance of identified cell populations between conditions (Fig. 2B). The most pronounced differences were observed in monocyte, T cell, and B cell repertoires. Non-classical monocyte cells were the least abundant in the Non-LC group. By contrast, the classical monocyte cell population was more prominent in the LC-Pulm group and even more prominent in the LC-CV group, suggesting a possible expansion of this population in patients who developed pulmonary or cardiovascular complications. Similarly, the proportion of immature CD4^+^ T cells in the population was more prominent in both complication groups compared with the Non-LC group. Immature and activated CD8^+^ T cell populations were most abundant in the Non-LC group. Comparably, the plasma B cell population was more prominent in the fully recovered Non-LC group. Interestingly, both classical dendritic cells (DCs) and plasmacytoid DCs were more abundant in the Non-LC group, with the lowest abundance in the LC-Pulm group.

Comparison of the relative number of cells in the identified populations revealed evident changes at the acute phase of COVID-19 and even 1.5–2 years post-infection across disease conditions (Fig. 2C). During the acute phase, a significant increase in the relative number of basophils was observed in both complication groups, with the LC-Pulm group showing the highest observed log_2_-fold increase. Conversely, erythroid-like cell numbers increased most notably in the LC-CV group, whereas this population notably decreased in the LC-Pulm group at the same time point. Significant increases were observed in non-classical monocytes, classical monocytes, and classical DCs in both study groups. B cell numbers were evidently higher in the LC-Pulm group, while the LC-CV patients showed a notable increase in NK cell and proliferating lymphocyte populations. A significant decrease in the relative number of immune cells during the acute phase was evident for multiple lymphoid cell populations in both Long-COVID complication groups. No notable differences in cell population proportions were observed in either of the complication groups for plasmacytoid DCs, pre-B cells, T regulatory cells (T_regs_), granulocytes, or platelets, or for CD4^+^ and proliferating lymphocyte subsets in the LC-Pulm group, or plasma B and cytotoxic T cells in the LC-CV group (Fig. 2C, right panel).

At 1.5–2 years post-acute infection, only a limited number of cell populations showed a considerable increase in the relative number of cells in the complication groups. Immature CD4^+^ T cell numbers were elevated in both complication groups, while erythroid-like cell numbers were increased exclusively in the LC-Pulm group. Long-COVID patients displayed a noticeable decline in plasma B cells and plasmacytoid DCs, with smaller reductions in B and pre-B cells, non-classical monocytes, erythroid-like cells, platelets, proliferating lymphocytes, activated CD8^+^ T cells, classical DCs, and basophils. No distinct differences in cell numbers at 1.5–2 years post-infection were found among several populations, including most lymphocytes and classical monocytes in the LC-CV group, and additionally platelets in the LC-Pulm group.

**Fig. 3:**
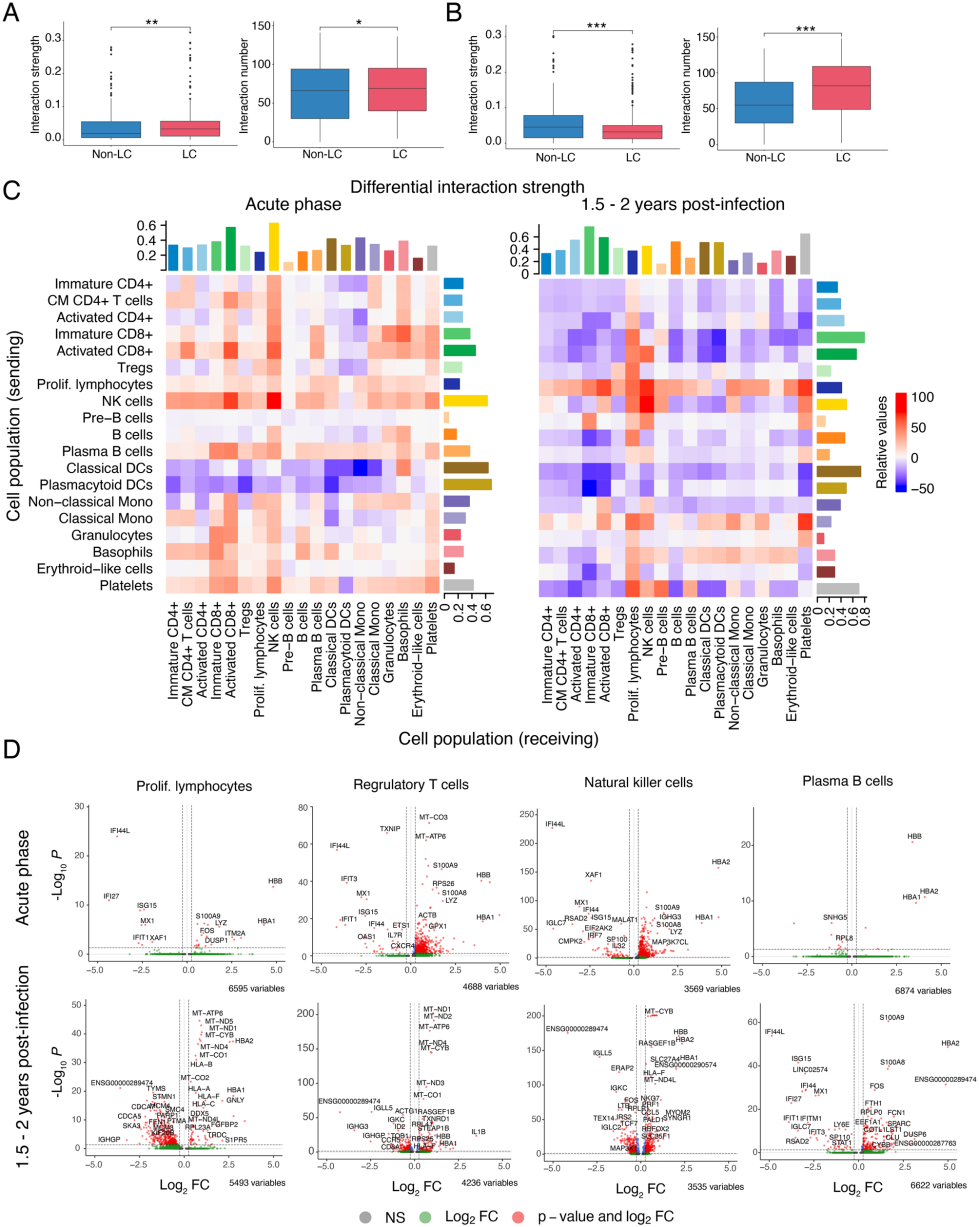
Intercellular communication between identified cell populations in recovered (Non-LC) and Long-COVID (LC) patient samples. (**A** and **B**) Total cell-to-cell interaction strength and the number of interactions inferred using the ligand–receptor CellChatDB Secreted Signalling database, between patients who developed Long-COVID (LC-CV and LC-Pulm groups) and patients who did not develop Long-COVID (Non-LC group) at the time of acute infection (**A**) and 1.5–2 years post-infection (**B**). (**C**) Heatmaps showing the differential interaction strength between previously identified cell populations. The y-axis represents the cell populations in which cells express the ligands (sending) from the CellChatDB Secreted Signalling database, and cell populations where cells express the corresponding receptor (receiving) are depicted on the x-axis. Differential interaction strength is compared between fully recovered (Non-LC, *n* = 3) and Long-COVID (LC-Pulm and LC-CV, *n* = 6) patients during the acute phase of infection and 1.5–2 years post-infection (**C**). Bar graphs summarize the total outgoing (sending, x-axis) and incoming (receiving, y-axis) differential interaction strength per cell population. (**D**) Differential gene expression (x = log_2_ Fold change, y = ˗log_10_P) between the Non-LC and LC groups in proliferating lymphocyte, T_reg_, NK, and plasma B cell populations at the time of acute infection and 1.5–2 years post-infection

### Patients with, and those that subsequently develop, Long-COVID exhibit perturbed intercellular communication and differentially expressed genes

Given the similarities in significantly altered immune cell population proportions in both the LC-CV and LC-Pulm groups relative to the controls (Fig. 2C), and considering the limited subgroup sizes, subsequent analyses were performed on the combined Long-COVID cohort to increase the statistical power for detecting shared transcriptional and interaction-level signatures. To investigate intercellular signalling networks in fully recovered (Non-LC) and Long-COVID (LC) patients, we performed CellChat analysis to infer ligand–receptor-based signalling interactions between immune cell populations. Differential interaction analysis was performed to evaluate absolute cell signalling in Non-LC and LC patients (Fig. 3A, B), showing both the total interaction strength and the number of interactions between the Non-LC and LC groups. The heatmap (Fig. 3C) depicts transmitted interaction strength from sender to receiver cell populations between conditions. The differential number of interactions at the acute infection phase and 1.5–2 years post-infection are shown in Fig. S4. Significant differences were observed in cell-to-cell interaction patterns, with LC patient groups (LC-Pulm and LC-CV) exhibiting an increased strength and number of interactions compared with the Non-LC group at the acute phase of COVID-19 infection (Fig. 3A). At 1.5–2 years post-infection, the LC groups showed a significant decrease in the overall cell-to-cell communication strength. By contrast, the number of interactions was significantly increased (Fig. 3B).

We further explored the observed decrease in interaction strength by individually analysing each of the previously identified cell populations, represented in Fig. 3C. High differential signalling strength was observed in immature and activated CD8^+^ T cells, classical DCs, and platelet populations, while the lowest differential signalling capacity was found in pre-B cells and granulocytes at 1.5–2 years post-infection (Figs. 3C, 1.5–2 years post-infection). Likewise, the highest differential signal-receiving capacity was found for immature cytotoxic T cells and platelets, while the lowest signal-receiving strength was found for pre-B cell and granulocyte populations. Although the interaction strength was generally decreased across most cell populations at 1.5–2 years post-infection for LC patients compared with Non-LC patients, proliferating lymphocytes, classical monocytes, NK cells, and basophils showed increased interactions with multiple other cell populations, emphasizing the connection between proliferating lymphocytes and NK cell groups. Proliferating lymphocytes in the LC group showed a consistent and robust increase in interaction strength with all receiving cell populations, except for plasmacytoid DCs, whereas classical monocytes showed increased interactions with all cell populations, except for plasma B cells.

During the acute phase of infection, the overall interaction strength was increased in the LC groups compared with the Non-LC group (Fig. 3C, acute phase). The greatest increase in outgoing signalling strength was observed for NK cells, the greatest decrease in outgoing signalling strength was observed for classical and plasmacytoid DCs, while the signal-receiving capacity was highest for activated CD8^+^ T and NK cell populations. Pre-B cells showed the least change in outgoing and receiving signal strength, which remained low at the post-infection stage, as observed previously. The strength of interactions at the acute phase was reduced for classical DCs and plasmacytoid DCs with all receiving cell populations, except for basophils. Moreover, there was an apparent decrease in interaction strength capacity between T cell subsets with DCs and non-classical monocytes.

Lastly, we compared differentially expressed genes in proliferating lymphocyte, T_reg_, NK, and plasma B cell populations between the Non-LC and LC groups (Fig. 3D). Haemoglobin-associated genes (*HBB*, *HBA1*, *HBA2*) remained significantly upregulated in all examined cell populations during both acute infection and the long-term follow-up phase. Similarly, S100 family genes (*S100A8*, *S100A9*) were consistently elevated across most cell types at both time points, indicating prolonged inflammatory activation.

By contrast, IFN-I-associated genes (*IFI44L*, *IFI44*, *IFI27*, *IFIT1*, *IFIT3*) and *ISG15* were significantly downregulated in LC patients during the acute phase but showed partial normalization at 1.5–2 years post-infection, suggesting early impairment of antiviral responses that does not persist uniformly across all compartments. Notably, additional transcriptional changes emerged during the chronic phase. T_reg_ cells demonstrated increased *IL1B* expression at 1.5–2 years post-infection, indicative of a late proinflammatory shift not evident during acute disease. Furthermore, proliferating lymphocytes in LC patients exhibited pronounced upregulation of HLA class I genes (*HLA-A*, *HLA-B*, *HLA-C*, *HLA-F*) specifically at the chronic phase time point, suggesting sustained or reactivated antigen presentation capacity in long-term disease. Together, these findings suggest that immune system dysfunction begins during the acute phase and continues up to 1.5–2 years after infection in patients with Long-COVID.

### Long-COVID patient lymphocyte subpopulations present with sustained cytotoxic activation and exhaustion-associated marker expression

**Fig. 4:**
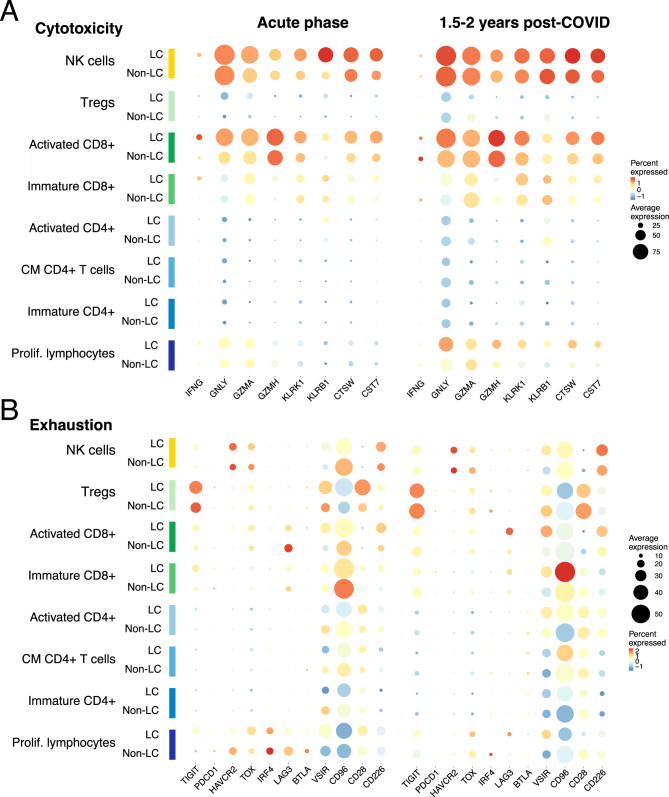
Cytotoxicity and exhaustion cell states. (**A** and **B**) The expression of well-established (**A**) cytotoxicity (*IFNG*, *GNLY*, *GZMA*, *GZMH*, *KLRK1*, *KLRB1*, *CTSW*, *CST7*) and (**B**) exhaustion-associated (*TIGIT*, *PDCD1*, *HAVCR2*, *TOX*, *IRF4*, *LAG3*, *BTLA*, *VSIR*, *CD96*, *CD28*, *CD226*) genes in T, NK, and proliferative lymphocyte subsets during the acute phase of infection and 1.5–2 years post-infection for the fully-recovered (Non-LC) and complication (LC) patient groups. Coloured cell populations and patient groups are represented on the y-axis, with genes of interest shown along the x-axis. Average gene expression is indicated as the dot size, and the dot colour denotes the percentage of cells in which the gene of interest was expressed

Next, we conducted an in-depth analysis of specific immune cell subsets to explore further the underlying mechanisms driving differential cell signalling patterns. We assessed the potential COVID-19-induced immune regulatory effects by examining the properties of the T, NK, and proliferative lymphocyte subsets. We compared the expression of well-established cytotoxicity (*IFNG*, *GNLY*, *GZMA*, *GZMH*, *KLRK1*, *KLRB1*, *CTSW*, *CST7*) (Fig. 4A) and exhaustion-associated (*TIGIT*, *PDCD1*, *HAVCR2*, *TOX*, *IRF4*, *LAG3*, *BTLA*, *VSIR*, *CD96*, *CD28*, *CD226*) (Fig. 4B) genes to describe cell state variance between fully recovered (Non-LC) and LC complication groups (LC-CV and LC-Pulm).

During the acute phase of infection, patients in the LC groups exhibited enhanced cytotoxic signatures across multiple lymphocyte subsets compared with patients in the Non-LC group. NK cells in the LC groups showed elevated expression of cytotoxicity-associated genes including *KLRB1* and *CST7*, while activated CD8^+^ T cells also displayed increased expression of *CST7* in addition to *CSTW*, indicating heightened cytotoxic potential early in infection. In parallel, T_reg_ cells from patients in the LC groups expressed higher levels of inhibitory receptors such as *TIGIT* and *VSIR*, suggesting early engagement of regulatory pathways. By contrast, certain activation-associated markers, including *CD96*, were more prominently expressed in immature CD8^+^ T cells in the Non-LC group during the acute phase, indicating differences in early immune activation between recovery trajectories.

Several of these cytotoxic programs persisted selectively in patients in the LC groups. Activated CD8^+^ T cells maintained elevated *CST7* and *CSTW* expression up to 1.5–2 years post-infection. Moreover, proliferative lymphocytes in patients in the LC groups demonstrated sustained upregulation of multiple cytotoxic markers, including *GNLY*, *GZMH*, *KLRK1*, *KLRB1*, *CST7*, and *CSTW*, at long-term follow-up. These findings indicate that enhanced cytotoxic activation in Long-COVID is not limited to the acute phase but extends into the chronic stage. Concurrently, exhaustion-associated signatures evolved over time in patients in the LC groups. Certain inhibitory receptors were already elevated during acute infection, whereas markers such as *LAG3* and *VSIR* became more pronounced in activated CD8^+^ T cells, proliferative lymphocytes, and NK cells at 1.5–2 years post-infection relative to Non-LC individuals. Together, these data suggest that Long-COVID is characterized by sustained cytotoxic activation coupled with persistent upregulation of inhibitory and exhaustion-associated pathways, reflecting incomplete immune resolution extending well beyond the acute phase.

### Persistent immune dysregulation in Long-COVID observed through altered interferon responses, inflammation, and cell migration

**Fig. 5:**
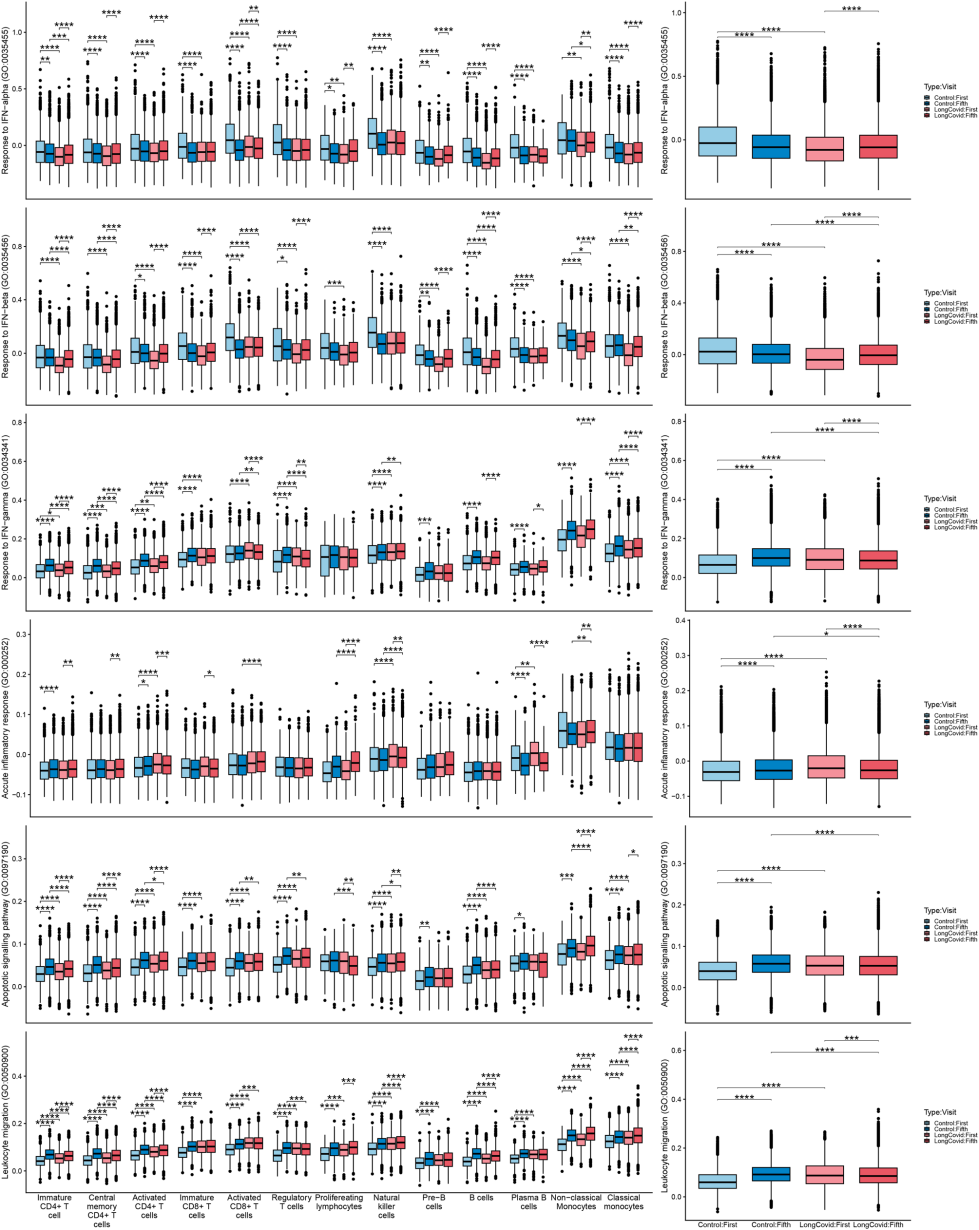
T, NK, B, and monocyte subpopulation cell state scores between conditions and time points. (**A** and **B**) Cell state scores calculated using the AddModuleScore function in Seurat indicating the IFN-α, -β, and -γ responses, the inflammatory response, apoptosis, and migration-associated gene expression in (**A**) each of the T, NK, B, and monocyte cell subpopulations, and (**B**) the total scores between the Non-LC and LC groups at the acute phase of the infection and at 1.5–2 years post-infection. Brackets denote significance from Dunn’s test with Holm-adjusted p-values (p.adj). Results are shown for combined and cell-type-specific groups. Significance: * p.adj < 0.05; ** < 0.01; *** < 0.001; **** < 0.0001

To further investigate the possible physiological implications of the previously observed cellular variations between the conditions, we calculated cell scores for the IFN-α, -β, and -γ responses, migration, and apoptosis-associated gene expression in T, NK, B, and monocyte cell subpopulations (Fig. 5A), and across the Non-LC and LC groups overall (Fig. 5B) at the acute and post-infection time points.

During the acute phase, IFN-α and -β responses were significantly higher in the Non-LC group across all analysed subpopulations, whereas the IFN-γ response was elevated in the LC groups, particularly in T, NK cells (*p* ≤ 0.0001), and classical monocytes (*p* ≤ 0.0001) (Fig. 5A), together indicating a stronger type II, but weaker type I, interferon (IFN-I) response in patients in the LC groups (Fig. 5B). Acute inflammatory response and apoptotic signalling associated gene expression levels were higher in several lymphocyte populations, while leukocyte migration was higher among all analysed cell subpopulations in the LC groups (Fig. 5A, B). Taken together, these findings point to a heightened inflammatory immune response in patients who later develop Long-COVID during the acute infection, suggesting early onset immune system perturbations.

At 1.5–2 years post-infection, the IFN-α response was higher in certain LC lymphocyte subpopulations in the LC groups, including immature CD4^+^ (*p* ≤ 0.001) and activated CD8^+^ (*p* ≤ 0.0001). However, the IFN-α response was lower in non-classical monocytes (*p* ≤ 0.05), and no significant differences were observed in the total IFN-α response between the study groups. By contrast, the IFN-β response remained lower for most subpopulations in the LC groups, except for activated CD8^+^ T cells. The IFN-γ response was generally reduced in the LC groups, apart from activated CD8^+^ T cells, where it remained elevated (*p* ≤ 0.01). The inflammatory response was persistently higher in the LC groups across multiple lymphoid cell types even 1.5–2 years post-infection. Furthermore, apoptotic signalling was lower in most lymphocyte populations in the LC groups, but remained significantly higher in NK cells (*p* ≤ 0.05) and non-classical monocytes (*p* ≤ 0.0001). Meanwhile, leukocyte migration was elevated (*p* ≤ 0.0001) in immature and activated CD4^+^, B cells, NK and monocyte subpopulations (Fig. 5A). Overall, the acute inflammatory response and apoptotic signalling were significantly lower, but leukocyte migration, and IFN-β and γ responses were higher in patients in the LC groups (Fig. 5B), indicating ongoing immune system activation 1.5–2 years post-infection.

The observed differences in apoptotic signalling pathways and leukocyte migration are further supported by data from a blood bulk transcriptome dataset derived from an extended patient cohort (Fig. S5). In recovered patients, apoptotic signalling was significantly higher (*p* ≤ 0.01) in the acute phase compared to 1.5–2 years post-diagnosis, whereas no significant changes were observed in patients with Long COVID. In contrast, leukocyte migration pathways showed a more pronounced reduction over time in Long COVID patients (*p* ≤ 0.01) compared with recovered individuals (*p* ≤ 0.05).

## Discussion

Here, we present a unique dataset with a detailed single-cell transcriptomic analysis of PBMCs in both acute and Long-COVID patients. Our findings illustrate shifts in the immune cell subpopulations between groups, cell communication alterations, and differential expression of genes with key functions. These collective findings provide insight into the immune landscape of COVID-19 as the disease progresses into a chronic, Long-COVID state. Several cell types were of particular interest, including proliferating lymphocytes, T cells, NK cells, monocytes, and B cells. The patterns identified indicated that distinct blood immune cell populations may play specific roles in the progression and persistence of Long-COVID.

Our analyses revealed cytotoxicity-associated gene expression in the proliferating lymphocytes and multiple T cell subsets of Long-COVID patients, which may exacerbate tissue damage and drive chronic inflammation. Previous research has also shown that cytotoxic activation is preserved several months to even a year after acute COVID-19 infection [17, 38]. Proliferating lymphocytes in patients in the Long-COVID group express cytotoxic markers, namely *GNLY*, *GZMH*, *KLRB1*, *CSTW*, and *CST7*, 1.5–2 years after acute COVID-19 infection, indicating a cytotoxic cell phenotype and potential involvement in the pathogenesis of chronic symptoms for an extended period. The apparent increased intercellular signalling patterns in proliferating lymphocytes, along with the upregulation of cytotoxicity-associated genes is suggestive of persistent immune activation, potentially linked to incomplete recovery from the acute infection.

Cytotoxic T cell subsets and proliferating lymphocytes in patients in the LC groups also exhibited higher expression of exhaustion-associated markers, such as *LAG3*, *TOX*, and *VSIR*, in comparison with the populations in patients who completely recovered, suggesting lasting immune regulation changes in Long-COVID patients. Yin et al. (2024) characterized the expression of exhaustion-associated markers in the T cells of COVID patients 8 months after the initial infection [17]. Although in our study we did not observe a high-level classical exhaustion marker expression pattern (*PD-1*, *CTLA*, *TIM-3*), we detected the expression of other immunomodulatory and co-stimulatory markers in the Long-COVID patient group up to 2 years after acute infection. Activated CD8^+^ T cells expressed *LAG3* in patients in the Non-LC group during acute infection, but the expression levels were higher 1.5–2 years after infection in patients with Long-COVID. A similar trend was seen for *CD96* expression in the immature CD8^+^ T cell population. In addition, 1.5–2 years after infection in Long-COVID patients, the proliferating lymphocyte population was found to express *TOX*, which is regarded as a depleted CD8^+^ T cell phenotype marker [39, 40]. These results further confirm the link between cell exhaustion and Long-COVID, especially in lymphocyte subpopulations [41]. Chu et al. (2025) reported that T cell exhaustion may be initiated early in acute COVID-19. They showed that exhaustion precursor cells, phenotypically and epigenetically similar to cells described in others chronic infections, are present at the start of acute infection [42]. Our observations, in line with previous findings, indicate immune anergy across Long-COVID patient lymphoid cell populations, emphasizing the necessity for further research on the long-term functional consequences. Together, exhaustion-associated gene expression, in addition to the cytotoxic profiles across lymphocyte populations, suggest immune system dysregulation in Long-COVID, highlighting an area for further exploration.

Chronic cell activation may lead to exhaustion, as observed in multiple long-term chronic illnesses. A continuous reaction to the presence of a permanent virus reservoir or to circulating SARS-CoV-2 residues may serve as potential triggers of immune system activation over an extended period, as observed in our study by the cytotoxic activation in several cell populations. Viral proteins and RNA have been detected in various tissues, including the liver and lungs [43, 44], and in various biological samples, such as plasma, urine, and faeces [45, 46]. In addition, research indicates that the circulating SARS-CoV-2 spike (S) protein is present in approximately 60% of patients with Long-COVID up to 12 months after acute infection [47]. Similar findings show viral presence in the gastrointestinal tract, which may indicate a long-term gastrointestinal tract cell infection with SARS-CoV-2 in Long-COVID patients [44–46, 48].

Chronic cytotoxic cell activation may hinder efficient recovery, maintaining inflammatory responses and thus promoting the onset of Long-COVID symptoms. Frere et al. (2022) reported a direct link between SARS-CoV-2 and tissue damage in a rodent model, which was consistent with observations in human tissues after COVID-19. The authors also noted T cell activation and distinct inflammatory cytokine and interferon reactions 1 month after COVID-19 infection, although the virus was not detected [49]. The evidence of HLA gene expression 1.5–2 years post-infection further confirms proliferating lymphocyte activation in Long-COVID patients. Previous research indicates that different HLA class I gene alleles correlate both with the likelihood of developing, and protection against, the severe course of COVID-19 [50–54]. Increased HLA class I gene expression almost 2 years after acute infection may reflect permanent antigen stimulation or autoimmune processes in Long-COVID patients. Research shows that SARS-CoV-2 has the potential to promote autoimmunity. Chang et al. (2023) reported that COVID-19 patients are at higher risk of developing autoimmune diseases [55]. After recovering from COVID-19, patient blood samples also showed elevated autoantibody levels, including antibodies against ACE2 [56]. Together these observations outline cytotoxic and proliferating lymphocytes as potential key modulators of Long-COVID complications.

Moreover, our analyses reveal distinct changes in the innate immune cell populations. Mononuclear phagocytes have been previously implicated in contributing to severe COVID-19 cases [57] and Long-COVID pathogenesis [58]. Both Long-COVID complication groups showed relatively larger monocyte populations at the acute phase, which, coupled with a high IFN-γ response, may indicate a necessity for functional cell recruitment in patients who develop Long-COVID. Conversely, a decrease in the non-classical monocyte population 1.5–2 years post-infection, along with lower IFN-α, -β, and -γ responses in the Long-COVID patient groups, signifies the involvement of innate immune system regulation. A reduction in non-classical monocyte populations during COVID-19 infection has also been observed [59]. Edahiro et al. (2023) proposed reduced transition potential from classical to non-classical monocytes in COVID-19, which could explain the changes in cell population proportions [57]. In addition, B cells showed a reduced ability to interact with multiple cell populations in Long-COVID patient groups, which raises concern about long-term adaptive immunity function. At the acute phase of infection, pre- and plasma B cells exhibit more prominent inflammatory responses, whereas at 1.5–2 years post-infection, B cell inflammatory properties (IFN-β and -γ responses) were lower in Long-COVID patients versus Non-LC patients. Interestingly, 1.5–2 years after infection, plasma B cells showed downregulation of IFN-I, as well as S100A8/A9 gene expression patterns were upregulated at the acute phase in other lymphocyte populations.

Upregulation of inflammatory S100 alarmins within lymphocyte populations suggests sustained innate immune activation in Long-COVID, which has been previously described in acute COVID-19 cases [60–62]. Notably, these transcriptional alterations persist up to 1.5–2 years post-infection, underscoring the long-term nature of Long-COVID. Haemoglobin-associated gene expression remains elevated in LC patients from the acute phase through to the long-term follow-up, consistent with previously described erythroid observations in acute COVID-19 [63]. Acute phase downregulation of IFN-I genes and *ISG15* indicate an early impairment of antiviral defence programs in individuals who subsequently develop Long-COVID [64, 65], while at later stages, increased *IL1B* expression in T_reg_ cells and enhanced HLA class I gene expression in proliferating lymphocytes suggest sustained proinflammatory signalling [66] and antigen presentation and may serve as potential determinants of disease severity [50–53]. Together, these findings support incomplete immune resolution in Long-COVID characterized by early antiviral reprogramming followed by chronic inflammatory and antigen-presenting activation.

During acute infection, all patients who subsequently developed Long-COVID showed reduced IFN-α and -β responses across the analysed cell populations. We also showed that plasmacytoid DCs have a high differential cell-to-cell interaction strength and low-level interactions with other cell populations, which is indicative of an impaired antiviral immune response at the acute infection stage. It should be noted that the reduced IFN-I reaction during acute infection in patients who subsequently develop Long-COVID confirms the hypothesis that an early, incomplete reaction against the virus can develop into a chronic, long-term condition [67]. Alongside the reduced IFN-I responses, the IFN-γ response was increased in Long-COVID patients at the acute stage. Several studies suggest that IFN-γ may be one of the possible mediators in the development of Long-COVID symptoms. Krishna et al. (2024) found that COVID-19 patient blood immune cell samples showed increased IFN-γ levels, which are mediated by CD8^+^ T cells [68]. In our study, higher *IFNG* expression during acute infection was observed in activated CD8^+^ cells in patients who subsequently developed Long-COVID. It is important to note that Li et al. (2024) identified IFN-γ as a potential driver in pulmonary complication development. They also reported that, in mice, anti-IFN-γ therapy after acute COVID-19 reduced the amount of lung tissue fibrosis [69].

Our single-cell RNA analyses provide a detailed overview of the population dynamics and molecular processes within blood immune cells; however, functional assays, flow cytometric analysis, and proteomics are needed to confirm these observed differences and determine therapeutic strategies to target Long-COVID. Our study has revealed interesting insight into the proliferating lymphocyte subpopulation, which now warrants independent exploration with more targeted approaches. One potential limitation of our study is that the sample size, although comprehensive, may not fully reflect the Long-COVID manifestations in patients with a more diverse range of symptoms, including neurological complications. However, our dataset is unique in its focus on a well-characterized cohort of initially hospitalized patients with prolonged Long-COVID, extending beyond the typical study periods and offering a rare insight into the long-term effects of this condition [70, 71]. It would be valuable to explore how the changes observed in PBMCs reflect broader systemic immune responses. To address this, we aim to integrate the results of this study with previously published gut microbiome data from patients within the same cohort [72] to facilitate ongoing research into the pathophysiology of Long-COVID. Using this approach, we aim to unravel the underlying mechanisms and systemic consequences of this condition.

It is essential to note that Long-COVID manifests with a variety of symptoms at different levels of severity, and issues regarding the classification and standardization of diagnoses may result in some cases remaining undetected. The interplay between Long-COVID symptoms and immune recovery remains poorly understood, with the determinants and triggers underlying the development of Long-COVID still under investigation [70]. This raises the possibility that while immune dysregulation is evident, it may not always translate into clinical diagnosis.

Taken together, this study shows distinct clustering of cell types, mitigated intercellular communication, and altered cell states in Long-COVID patients. Immune cell dysregulation in Long-COVID is evident in our findings and previous studies [17, 20, 58]. Differentially expressed genes and the involvement of innate immune system regulation, though widely documented in severe acute COVID-19 cases, may play a role in the development or persistence of Long-COVID, as highlighted by the outcomes of this investigation. The detailed comparison of cell states, populations, and ligand–receptor signalling provides new insight into the immune processes involved in acute and Long-COVID.

## Conclusions

Our findings reveal that Long-COVID manifests as an unresolved immune state, driven by ongoing cytotoxicity, interferon-mediated inflammation, and impaired cellular communication. Along with highlighting the molecular and cellular mechanisms involved, our study shows that immune dysfunction originates during the acute phase of infection and persists up to 1.5–2 years post-infection. At the time of acute infection, patients who developed Long-COVID exhibited a significantly impaired IFN response, evidenced by the downregulation of associated genes. Lymphocyte cell exhaustion-associated markers and cellular communication changes 1.5–2 years post-infection suggest that specific immune cell subsets play distinct roles in maintaining the proinflammatory environment characteristic of prolonged Long-COVID. Prolonged Long-COVID patients exhibit a marked reduction in overall intercellular signalling strength, particularly among lymphocyte populations. This study reinforces the notion that Long-COVID is a condition of sustained immune dysfunction, in which persistent immune activation likely contributes to tissue damage and multisystemic symptoms, including pulmonary and cardiovascular complications.

## Electronic supplementary material

Below is the link to the electronic supplementary material.

Supplementary material 1

## Data Availability

ScRNA-seq data have been submitted at the Sequence Read Archive (PRJNA1208100) and are in the process of depositing at Gene Expression Omnibus.
