# Supplementary material for: Immune dysregulation in prolonged Long-COVID: lymphocytes emerge as key mediators of persistent inflammation, exhaustion and cytotoxicity
Source: J Transl Med. 2026 Apr 11;24:527. doi: 10.1186/s12967-026-08081-6 (PMC13088835; doi:10.1186/s12967-026-08081-6)
Supplement: Supplementary file 1 — Supplementary material 1 [file 12967_2026_8081_MOESM1_ESM.docx]

**Immune dysregulation in prolonged Long-COVID: lymphocytes emerge as key mediators of persistent inflammation, exhaustion and cytotoxicity**

Marta Liva Springe^a,b^, Kristīne Vaivode^a,b^, Rihards Saksis^a^, Nineļa Miriama Vainšeļbauma^a^, Laura Ansone^a^, Monta Brīvība^a^, Helvijs Niedra^a^, Vita Rovite^a^*

*^a^Latvian Biomedical Research and Study Centre, LV-1067 Riga, Latvia.*

*^b^These authors contributed equally.*

*Corresponding author: Dr Vita Rovite

Email: vita.rovite@biomed.lu.lv; Tel. no.: +371 28474184

# Supplementary material

## Table of Contents

Figure S1 1

Figure S2 2

Figure S3 3

Figure S4 4

Figure S5 5

Table S1 6

Table S2 8

**
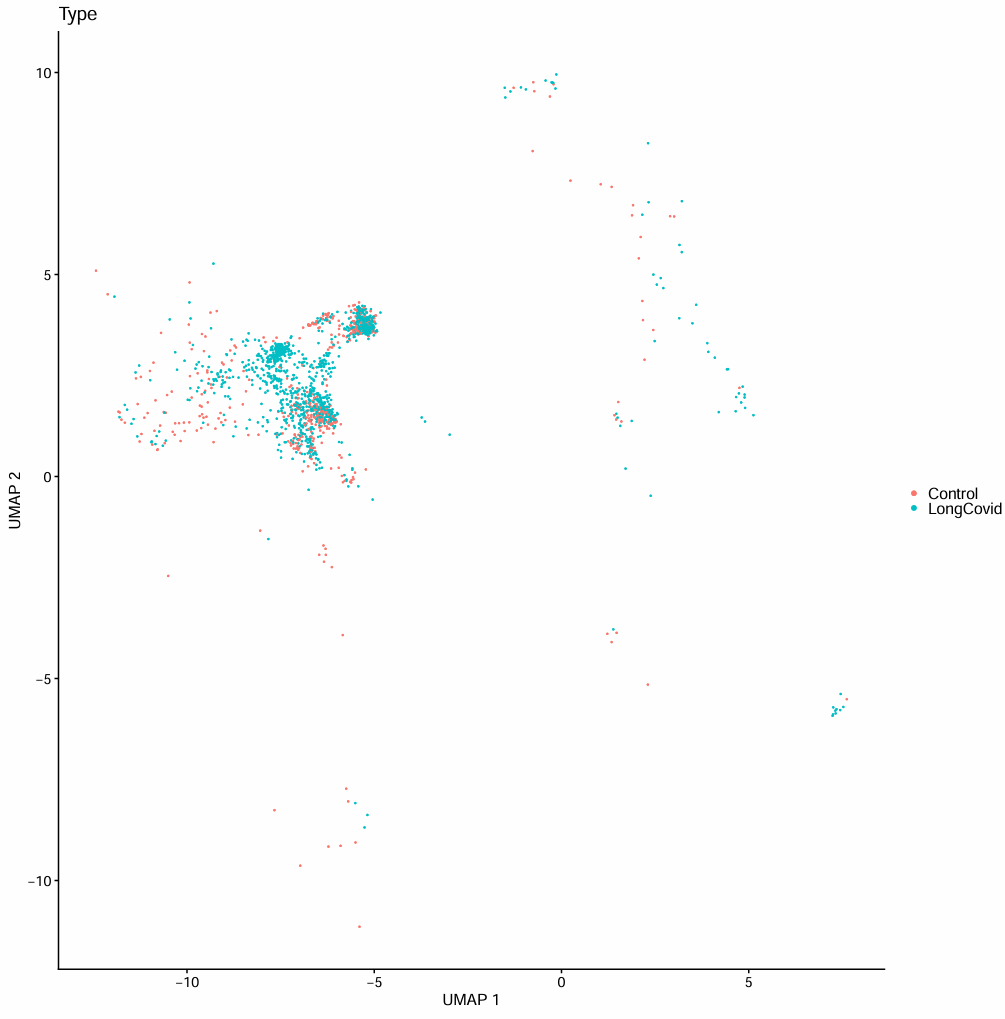
**

**Figure S1. Proliferative lymphocyte clustering between recovered and Long-COVID patients across all time points.**

**
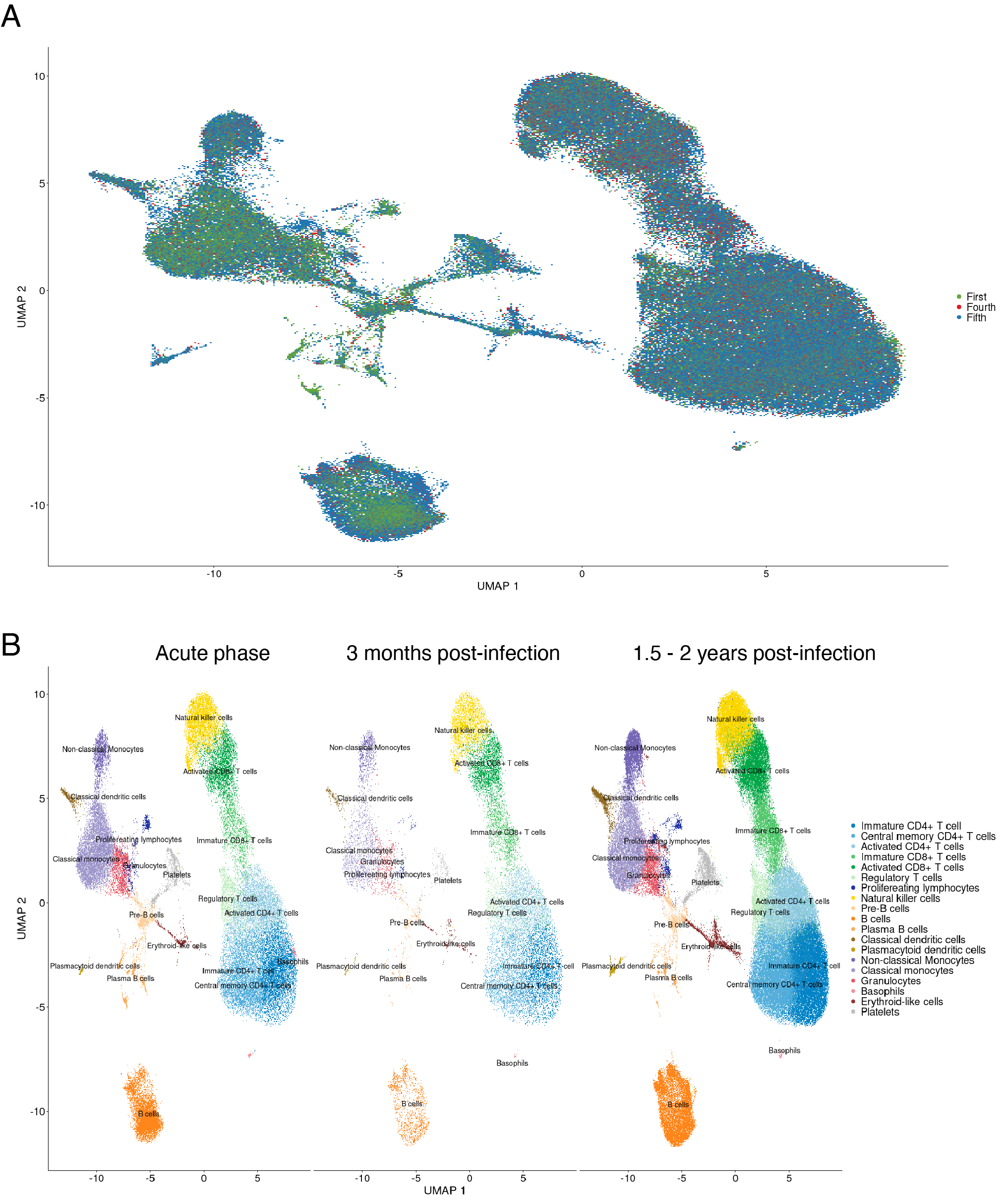
**

**Figure S2. Immune cell landscape at acute COVID-19 infection, 3 months and 1.5 – 2 years post-infection.**

(A) UMAP overlay of all patient cells at the three timepoints: acute COVID-19 infection, 3 months and 1.5 – 2 years post-infection.

(B) UMAP of the all patient cells at the three timepoints: acute COVID-19 infection, 3 months and 1.5 – 2 years post-infection.


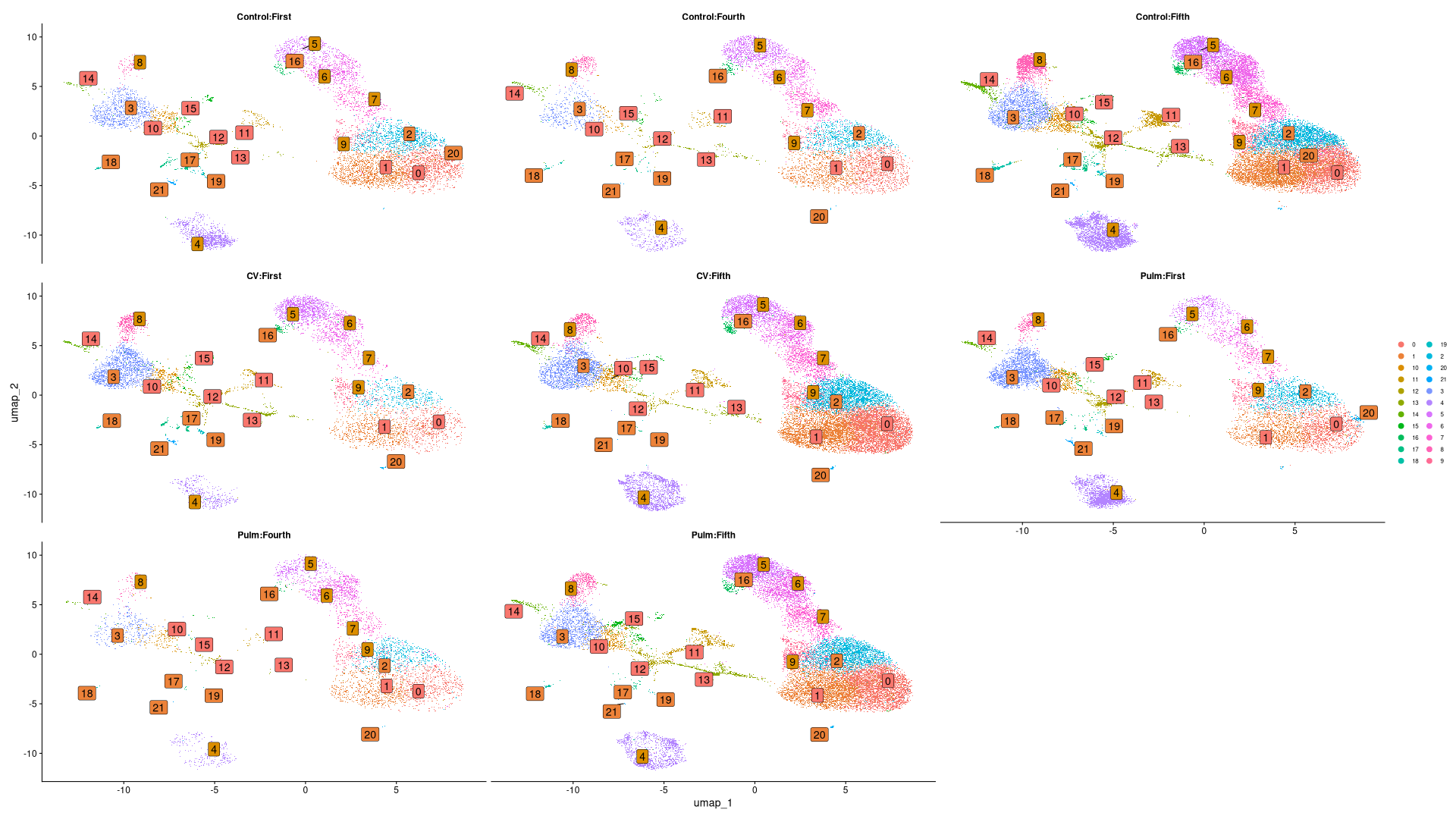


**Figure S3. Representation of the immune cell UMAP for each of the patient groups at acute COVID-19 infection, 3 months and 1.5 – 2 years post-infection.**


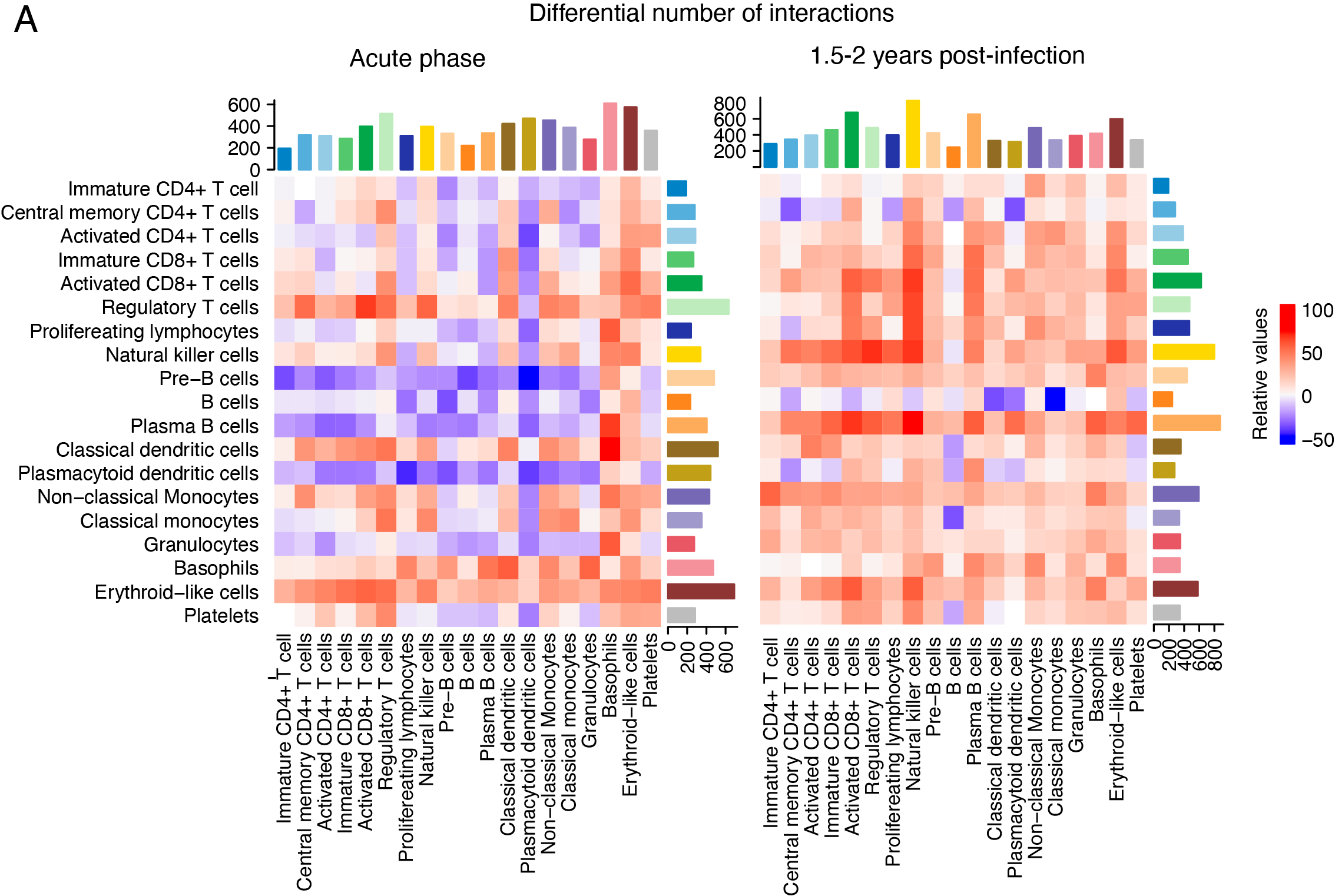


**Figure S4. Cell-to-cell differential number of interactions between conditions at the acute phase and 1.5 – 2 years post-infection.**

(A) Heatmaps showing the differential number of interactions and in the identified cell populations, compared between fully recovered (Non-LC, n = 3) and Long-COVID (LC-Pulm and LC-CV, n = 6) patients during the acute phase of infection and 1.5 – 2 years post-infection. Cell populations expressing the ligand are shown on the x-axis (sending) and the cell populations expressing corresponding the receptor (receiving) are shown on the y-axis.

**
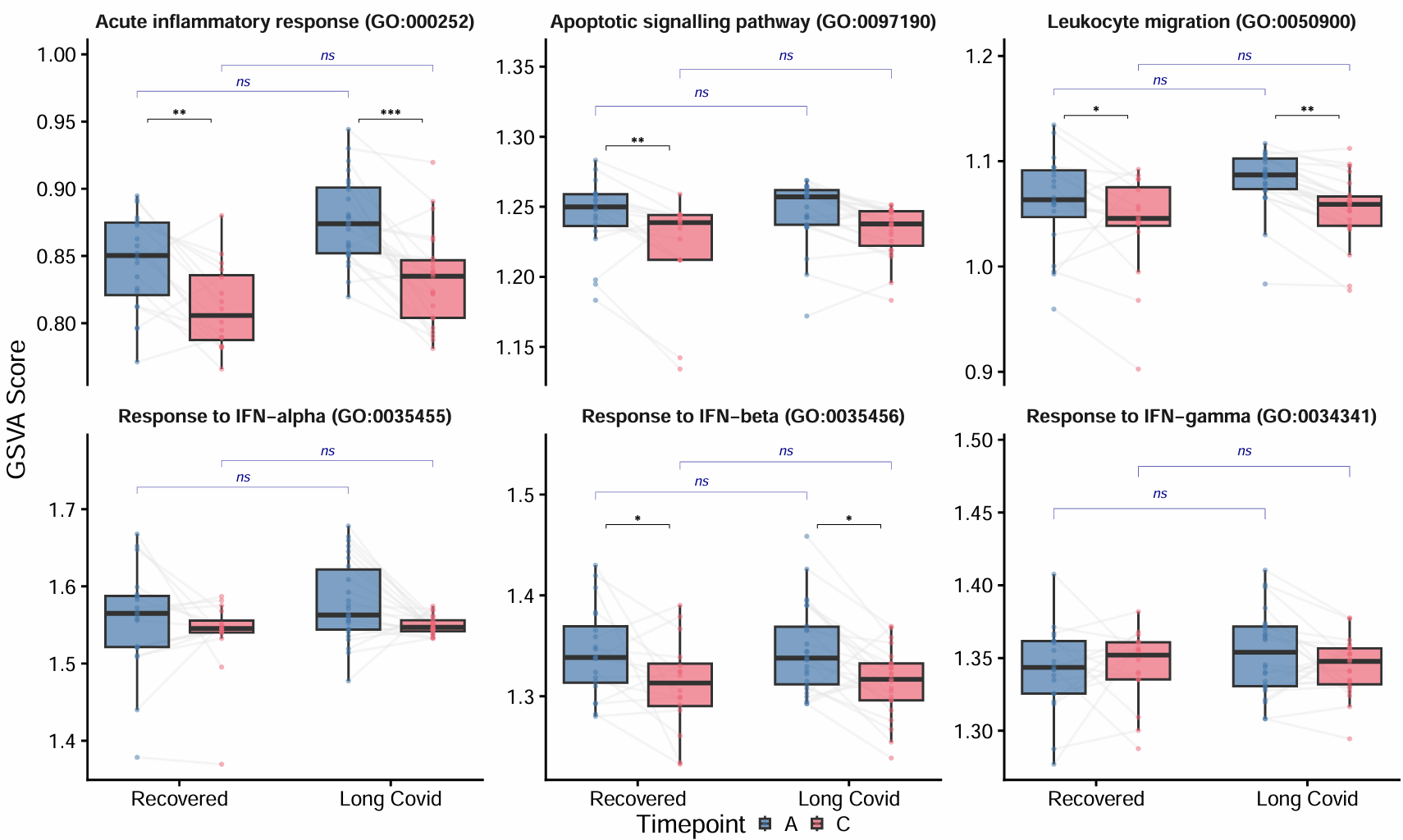
**

**Figure S5. Representation of the immune cell UMAP for each of the patient groups at acute COVID-19 infection, 3 months and 1.5 – 2 years post-infection.**

**Table S1. Patient cohort characteristics.**

| Patient | Study group | Age | Body mass index (BMI) | Vaccination (in order) | Medication during the acute phase of infection | How many times had COVID-19 |
| --- | --- | --- | --- | --- | --- | --- |
| P711 | LC-Pulm | 45 | 26,83 | Pfizer-BioNTech  Pfizer-BioNTech | Paracetamol  Diclofenac  Codeine  Ceftriaxone  Doxycycline  Chloropyramine hydrochloride  *Sirupus Pini Compositus*  Dexamethasone  Enoxaparin  Ringer's solution | 2 |
| P921 | LC-Pulm | 52 | 43,75 | Pfizer-BioNTech  Pfizer-BioNTech  Pfizer-BioNTech  Pfizer-BioNTech | N/A |  |
| P908 | LC-Pulm | 66 | 33,09 | N/A | N/A | N/A |
| P713 | Non-LC | 47 | 27,72 | Pfizer-BioNTech  Pfizer-BioNTech  Pfizer-BioNTech | Paracetamol  Codeine  Doxycycline  Bromhexine  Enoxaparin  Ascorbic Acid 10%  Omeprazole | 1 |
| P865 | Non-LC | 46 | 28,52 | Pfizer-BioNTech  Pfizer-BioNTech | Paracetamol  Ceftriaxone  Doxycycline  Remdesivir  Bromhexine  Enoxaparin  Ascorbic Acid 10%  Metoclopramide  Pancreatin  Neurorubine | 1 |
| P407 | Non-LC | 49 | 20,45 | Pfizer-BioNTech  Moderna | N/A | 1 |
| P841 | LC-CV | 55 | 28,34 | YES | Oxygen supplementation  Analgin  Ceftriaxone  Doxycycline  Remdesivir  Bromhexine  Dexamethasone  Enoxaparin  Ascorbic Acid 10%  Syr. Lactulose | 3 |
| P843 | LC-CV | 68 | 21,45 | NO | Oxygen supplementation  Analgin  Paracetamol  Ceftriaxone  Doxycycline 1  Bromhexine  Dexamethasone 8  Enoxaparin  Ascorbic Acid 10%  Omeprazole  Bromazepam | 2 |
| P854 | LC-CV | 71 | 23,88 | Janssen | Oxygen supplementation  Ceftriaxone  Doxycycline  Bromhexine  Dexamethasone  Enoxaparin  Ascorbic Acid 10%  *Mixturae nervinae*  *Sirupus Pini Compositus*  Quetiapine  Omeprazole | 1 |

**Table S2. Patient medical events and diagnosis during the time of the study.**

| Patient ID | Primary diagnosis (ICD-10 code) | Primary diagnosis (ICD-10 description) | Adjacent diagnosis (ICD-10 code) | Adjacent diagnosis (ICD-10 description) |
| --- | --- | --- | --- | --- |
| P407 | U07.1 | COVID-19, virus identified |  |  |
|  | Z20.8 | Contact with and exposure to other communicable diseases |  |  |
|  | U11.9 | Need for immunization against COVID-19, unspecified |  |  |

| **P711** | N95 | Menopausal and female climacteric states |  |  |
| --- | --- | --- | --- | --- |
|  | Z20.8 | Contact with and exposure to other communicable diseases |  |  |
|  | U07.1 | COVID-19, virus identified | J17 | Pneumonia in diseases classified elsewhere |
|  | M47 | Spondylosis |  |  |
|  | U11.9 | Need for immunization against COVID-19, unspecified |  |  |
|  | M19.9 | Arthrosis, unspecified | M47.2 | Other spondylosis with radiculopathy |
|  | M47.2 | Other spondylosis with radiculopathy | F33, G43.2 | Recurrent depressive disorder, current episode mild, *Status migrainosus* |
|  | R10 | Pain localized to upper abdomen |  |  |
|  | Z01.4 | Gynaecological examination (general)(routine) |  |  |
|  | Z12.4 | Special screening examination for neoplasm of cervix |  |  |
|  | U09 | Post COVID-19 condition |  |  |
|  | U07.1 | COVID-19, virus identified | B01.2 | Varicella pneumonia |
|  | I47.1 | Supraventricular tachycardia | U09.9 | Post COVID-19 condition, unspecified |
|  | S06.0 | Sequelae of intracranial injury | G43.2 | *Status migrainosus* |
| **P713** | Z20.8 | Contact with and exposure to other communicable diseases |  |  |
|  | U07.1 | COVID-19, virus identified |  |  |
|  | U11.9 | Need for immunization against COVID-19, unspecified |  |  |
|  | E03.8 | Other specified hypothyroidism | E78.2 | Mixed hyperlipidaemia |
|  | Z00.0 | General medical examination |  |  |
|  | Z03.8 | Observation for other suspected diseases and conditions |  |  |
|  | E03.8 | Other specified hypothyroidism | E79 | Disorders of purine and pyrimidine metabolism |
|  | J18 | Pneumonia, organism unspecified |  |  |
| **P841** | U07.1 | COVID-19, virus identified |  |  |
|  | Z20.8 | Contact with and exposure to other communicable diseases |  |  |
|  | N18.3 | Chronic kidney disease, stage 3 |  |  |
|  | U11.9 | Need for immunization against COVID-19, unspecified |  |  |
|  | J18.9 | Pneumonia, unspecified |  |  |
|  | L82 | Seborrhoeic keratosis | D18.0 | Haemangioma, any site |
|  | Z00.0 | General medical examination |  |  |
|  | Z03.8 | Observation for other suspected diseases and conditions |  |  |
|  | N30 | Acute cystitis |  |  |
|  | Z12.3 | Special screening examination for neoplasm of breast |  |  |
|  | Z12.3 | Special screening examination for neoplasm of breast |  |  |
|  | E66.0 | Obesity due to excess calories |  |  |
|  | U07.1 | COVID-19, virus identified | J18.9 | Pneumonia, unspecified |
|  | J20 | Acute bronchitis |  |  |
|  | R05 | Cough |  |  |
| **P843** | Z20.8 | Contact with and exposure to other communicable diseases |  |  |
|  | U07.1 | COVID-19, virus identified |  |  |
|  | D25 | Leiomyoma of uterus |  |  |
|  | D25.1 | Intramural leiomyoma of uterus |  |  |
|  | Z96.6 | Presence of orthopaedic joint implants |  |  |
|  | T93.1 | Sequelae of fracture of femur | Z96.6 | Presence of orthopaedic joint implants |
|  | Z13 | Special screening examination for other diseases and disorders |  |  |
|  | H43.2 | Crystalline deposits in vitreous body |  |  |
|  | A09.9 | Gastroenteritis and colitis of unspecified origin |  |  |
|  | T93.1 | Sequelae of fracture of femur | U07.9, Z96.6 | Presence of orthopaedic joint implants |
|  | J45.8 | Mixed asthma | I10, L40.8 | Essential (primary) hypertension, Other psoriasis |
|  | M16 | Coxarthrosis [arthrosis of hip] |  |  |
| **P854** | K31.0 | Acute dilatation of stomach |  |  |
|  | E06.3 | Autoimmune thyroiditis | D51, E03.8, M47 | Vitamin B12 deficiency anaemia, Other specified hypothyroidism, Spondylosis, unspecified |
|  | Z20.8 | Contact with and exposure to other communicable diseases |  |  |
|  | U07.1 | COVID-19, virus identified |  |  |
|  | E03 | Other hypothyroidism |  |  |
|  | K31.0 | Acute dilatation of stomach |  |  |
|  | E03.8 | Other specified hypothyroidism | E21, E78 | Hyperparathyroidism and other disorders of parathyroid gland, Disorders of lipoprotein metabolism and other lipidaemias |
|  | E03.8 | Other specified hypothyroidism | E21.3 | Hyperparathyroidism, unspecified |
|  | E04 | Other nontoxic goitre |  |  |
|  | M47 | Spondylosis |  |  |
|  | D51 | Vitamin B12 deficiency anaemia |  |  |
|  | E03.8 | Other specified hypothyroidism | E21.3, M82.1 | Hyperparathyroidism, unspecified, Osteoporosis in endocrine disorders |
|  | E04.2 | Nontoxic multinodular goitre | Z00.0 | General medical examination |
|  | F45.9 | Somatoform disorder, unspecified |  |  |
|  | J12.8 | Other viral pneumonia | U07.1 | COVID-19, virus identified |
|  | E03.8 | Other specified hypothyroidism |  |  |
|  | Z00.0 | General medical examination |  |  |
|  | M47.2 | Other spondylosis with radiculopathy |  |  |
|  | E03.8 | Other specified hypothyroidism | E78 | Disorders of lipoprotein metabolism and other lipidaemias |
|  | I80.1 | Phlebitis and thrombophlebitis of femoral vein |  |  |
|  | U09 | Post COVID-19 condition |  |  |
| **P865** | B00.1 | Herpesviral vesicular dermatitis | F45.3 | Somatoform autonomic dysfunction |
|  | U07.1 | COVID-19, virus identified |  |  |
|  | U11.9 | Need for immunization against COVID-19, unspecified |  |  |
|  | E03 | Other hypothyroidism |  |  |
|  | Z20.8 | Contact with and exposure to other communicable diseases |  |  |
|  | J06.9 | Acute upper respiratory infection, unspecified | I10 | Essential (primary) hypertension |
|  | Z03.8 | Observation for other suspected diseases and conditions |  |  |
| **P886** | Z20.8 | Contact with and exposure to other communicable diseases |  |  |
|  | U07.1 | COVID-19, virus identified |  |  |
|  | U09.9 | Post COVID-19 condition, unspecified |  |  |
|  | J06 | Acute upper respiratory infections of multiple and unspecified sites |  |  |
|  | N64 | Other disorders of breast |  |  |
|  | J15 | Bacterial pneumonia, not elsewhere classified |  |  |
|  | Z26 | Need for immunization against other single infectious diseases |  |  |
|  | Z27.8 | Need for immunization against other combinations of infectious diseases |  |  |
|  | U11.9 | Need for immunization against COVID-19, unspecified |  |  |
| **P908** | D25 | Leiomyoma of uterus |  |  |
|  | U09.9 | Post COVID-19 condition, unspecified |  |  |
|  | U11.9 | Need for immunization against COVID-19, unspecified |  |  |
|  | I10 | Essential (primary) hypertension |  |  |
|  | Z12.3 | Special screening examination for neoplasm of breast |  |  |
|  | E03.8 | Other specified hypothyroidism |  |  |
|  | G93.3 | Postviral fatigue syndrome | E03, I10 | Other hypothyroidism, Essential (primary) hypertension |
|  | Z20.8 | Contact with and exposure to other communicable diseases |  |  |
|  | E03.8 | Other specified hypothyroidism | E21.3 | Hyperparathyroidism, unspecified |
|  | H81.8 | Other disorders of vestibular function |  |  |
|  | Z20.8 | Contact with and exposure to other communicable diseases |  |  |
|  | U09.9 | Post COVID-19 condition, unspecified |  |  |
|  | G93 | Other disorders of brain |  |  |
|  | I10 | Essential (primary) hypertension |  |  |
|  | N73 | Other female pelvic inflammatory diseases |  |  |
|  | D18 | Haemangioma and lymphangioma, any site |  |  |
|  | Z12.1 | Special screening examination for neoplasm of intestinal tract |  |  |
|  | U07.2 | COVID-19, virus not identified |  |  |
|  | H25.8 | Other senile cataract | H43.3, H52.0, H52.4 | Other vitreous opacities, Hypermetropia, Presbyopia |
|  | J00 | Acute nasopharyngitis [common cold] | I10, L57.0, M47, Z12.1 | Essential (primary) hypertension, Actinic keratosis, Spondylosis, Special screening examination for neoplasm of intestinal tract |
| **P921** | U11.9 | Need for immunization against COVID-19, unspecified |  |  |
|  | J20 | Acute bronchitis |  |  |
|  | Z20.8 | Contact with and exposure to other communicable diseases |  |  |
|  | E11.7 | Type 2 diabetes mellitus with multiple complications |  |  |
|  | U07.1 | COVID-19, virus identified |  |  |
|  | U08 | Personal history of COVID-19 |  |  |
|  | U11.9 | Need for immunization against COVID-19, unspecified | E11.8 | Type 2 diabetes mellitus with unspecified complications |
|  | E11.7 | Type 2 diabetes mellitus with multiple complications | E06.3 | Autoimmune thyroiditis |
|  | Z25.1 | Need for immunization against influenza | E11.8 | Type 2 diabetes mellitus with unspecified complications |
|  | I10 | Essential (primary) hypertension | E11.7 | Type 2 diabetes mellitus with multiple complications |
|  | E11.4 | Type 2 diabetes mellitus with neurological complications | I10 | Essential (primary) hypertension |
|  | E11.7 | Type 2 diabetes mellitus with multiple complications |  |  |
|  | E11.7 | Type 2 diabetes mellitus with multiple complications | E78 | Disorders of lipoprotein metabolism and other lipidaemias |
|  | J18.9 | Pneumonia, unspecified | E11.8, I10 | Type 2 diabetes mellitus with unspecified complications, Essential (primary) hypertension |
|  | J06.9 | Acute upper respiratory infection, unspecified | D50.9, E11.7 | Iron deficiency anaemia, unspecified, Type 2 diabetes mellitus with multiple complications |
|  | R05 | Cough |  |  |
|  | Z20.8 | Contact with and exposure to other communicable diseases |  |  |
|  | J06.9 | Acute upper respiratory infection, unspecified | E11.4 | Type 2 diabetes mellitus with neurological complications |
|  | E11.4 | Type 2 diabetes mellitus with neurological complications | E06.3, I10, U08.9 | Autoimmune thyroiditis, Essential (primary) hypertension, Personal history of COVID-19, unspecified |
